# Strawberry fatty acyl glycosides enhance disease protection, have antibiotic activity and stimulate plant growth

**DOI:** 10.1038/s41598-020-65125-7

**Published:** 2020-05-18

**Authors:** Carlos Grellet Bournonville, María Paula Filippone, Pía de los Ángeles Di Peto, María Fernanda Trejo, Alicia Susana Couto, Alicia Mamaní de Marchese, Juan Carlos Díaz Ricci, Björn Welin, Atilio Pedro Castagnaro

**Affiliations:** 1 Instituto de Tecnología Agroindustrial del Noroeste Argentino (ITANOA), Estación Experimental Agroindustrial Obispo Colombres (EEAOC)–Consejo Nacional de Investigaciones Científicas y Técnicas (CONICET) (P.C. T4101), Las Talitas Tucumán, Argentina; 2 Centro de Investigaciones en Hidratos de Carbono, Departamento de Química Orgánica, Facultad de Ciencias Exactas y Naturales, Universidad de Buenos Aires (P.C. C1428), Buenos Aires, Argentina; 3 Facultad de Agronomía y Zootecnia, Universidad Nacional de Tucumán (UNT), San Miguel de Tucumán (P.C. T4000), Tucumán, Argentina; 4 Instituto Superior de Investigaciones Biológicas (INSIBIO, CONICET-UNT) and Instituto de Química Biológica “Dr. Bernabé Bloj”, Facultad de Bioquímica, Química y Farmacia, UNT, San Miguel de Tucumán (P.C. T4000), Tucumán, Argentina

**Keywords:** Field trials, Effectors in plant pathology, Plant signalling

## Abstract

An increasing interest in the development of products of natural origin for crop disease and pest control has emerged in the last decade. Here we introduce a new family of strawberry acyl glycosides (SAGs) formed by a trisaccharide (GalNAc-GalNAc-Glc) and a monounsaturated fatty acid of 6 to 12 carbon atoms linked to the glucose unit. Application of SAGs to *Arabidopsis thaliana* (hereafter Arabidopsis) plants triggered a transient oxidative burst, callose deposition and defense gene expression, accompanied by increased protection against two phytopathogens, *Pseudomonas viridiflava* and *Botrytis cinerea*. SAGs-induced disease protection was also demonstrated in soybean infected with the causal agent of target spot, *Corynespora cassiicola*. SAGs were shown to exhibit important antimicrobial activity against a wide-range of bacterial and fungal phytopathogens, most probably through membrane destabilization, and the potential use of SAGs as a biofungicide for postharvest disease protection was demonstrated on lemon fruits infected with *Penicillium digitatum*. Plant growth promotion by application of SAGs was shown by augmented primary root elongation, secondary roots development and increased siliques formation in Arabidopsis, whereas a significant increment in number of seed pods was demonstrated in soybean. Stimulation of radicle development and the induction of an auxin-responsive reporter system (DR5::GUS) in transgenic Arabidopsis plants, suggested that SAGs-stimulated growth at least partly acts through the auxin response pathway. These results indicate that strawberry fatty acid glycosides are promising candidates for the development of environmental-friendly products for disease management in soybean and lemon.

## Introduction

The excessive use of synthetic pesticides and fertilizers brought about by the green revolution has in many cases had a detrimental effect on the environment and has caused important negative effects on human and animal health. As a consequence stricter regulations have been implemented in many countries to reduce the number of synthetic agrochemicals available in agriculture to promote a more sustainable production system. Due to these recent changes in the limitation of the use of chemicals in agricultural production there is an urgent need to find effective and more environmental-friendly alternatives to significantly reduce and/or replace these products^1,2^. One sustainable strategy that has gained increasing interest in the last decades has been the development of integrated crop management programs where a combined use of non-chemical tools, including crop rotation, planting time, tillage, use of trap crops etc. and biological products are implemented^3–5^.

A lot of attention has been focused on the development of biostimulants and biofertilizers, supplied to the crop soil or applied directly on the plant, to improve crop yield size and quality by enhancing plant nutrition, reduce abiotic stress impact and promote plant growth^4,6,7^. In addition, biocontrollers or biopesticides have been developed for pest and disease control in many crops^8,9^. All these products of biological origin are more readily degradable and less persistent than chemical fertilizers and pesticides, which make them amenable as replacement for traditional agrochemicals in a sustainable crop production system.

Biocontrol products based on plant extracts with antimicrobial activity or microbial antagonists of pathogens have been implemented as an alternative method in integrated pest management for some time. More recently, defense elicitors that induce an incomplete, systemic resistance to a broad range of plant pathogens^10–13^, have gained a lot of interest. There is a vast variety of elicitors described both of natural and synthetic origin, which have been classified into two major groups; molecules that are or mimic phytohormones and compounds that mock the presence of a plant enemy^14^. Recently, various products have reached the market for disease control represented by both these groups of molecules^15^.

Fatty acyl glucosides are amphipathic compounds composed of a glycosyl moiety linked to one or more hydroxyl fatty acids or to one carboxyl group of a fatty acid by an ester linkage. These compounds are mainly produced by bacteria, yeast, fungi, marine invertebrates, and plants^16^. Because of their physicochemical and biological properties, they are used as surfactants, antibiotics and drugs in the oil, food, cosmetic and pharmaceutical industries^17,18^. Industrial interest in fatty acyl glycosides of natural origin (biosurfactants) has recently increased due to their low toxicity, high biodegradability, foaming capacity, high selectivity and specificity at extreme temperatures, pH and salinity.

Fatty acyl glucosides have been characterized in numerous plant species belonging to *Solanaceae*^19^ and in a few members of the *Caryophyllaceae*^20^, *Martyniaceae*^21^ and *Rubiaceae* families^22^. Studies conducted under controlled and field growing conditions have demonstrated that they play an important role in plant-insect and plant-fungus interactions^23–27^.

Previous works in our laboratory have demonstrated the capacity of different strawberry extracts and metabolites to protect plants against pathogen infections through enhanced plant immunity and by direct antimicrobial activity^28–31^. In this study, we present a group of strawberry trisaccharide fatty acid esters, not previously described, which activate innate plant pathogen defense reactions, stimulate plant growth and exhibit important antimicrobial activity.

## Results and discussion

### Bioassay-guided purification and chemical structure determination of SAGs

**Figure 1:**
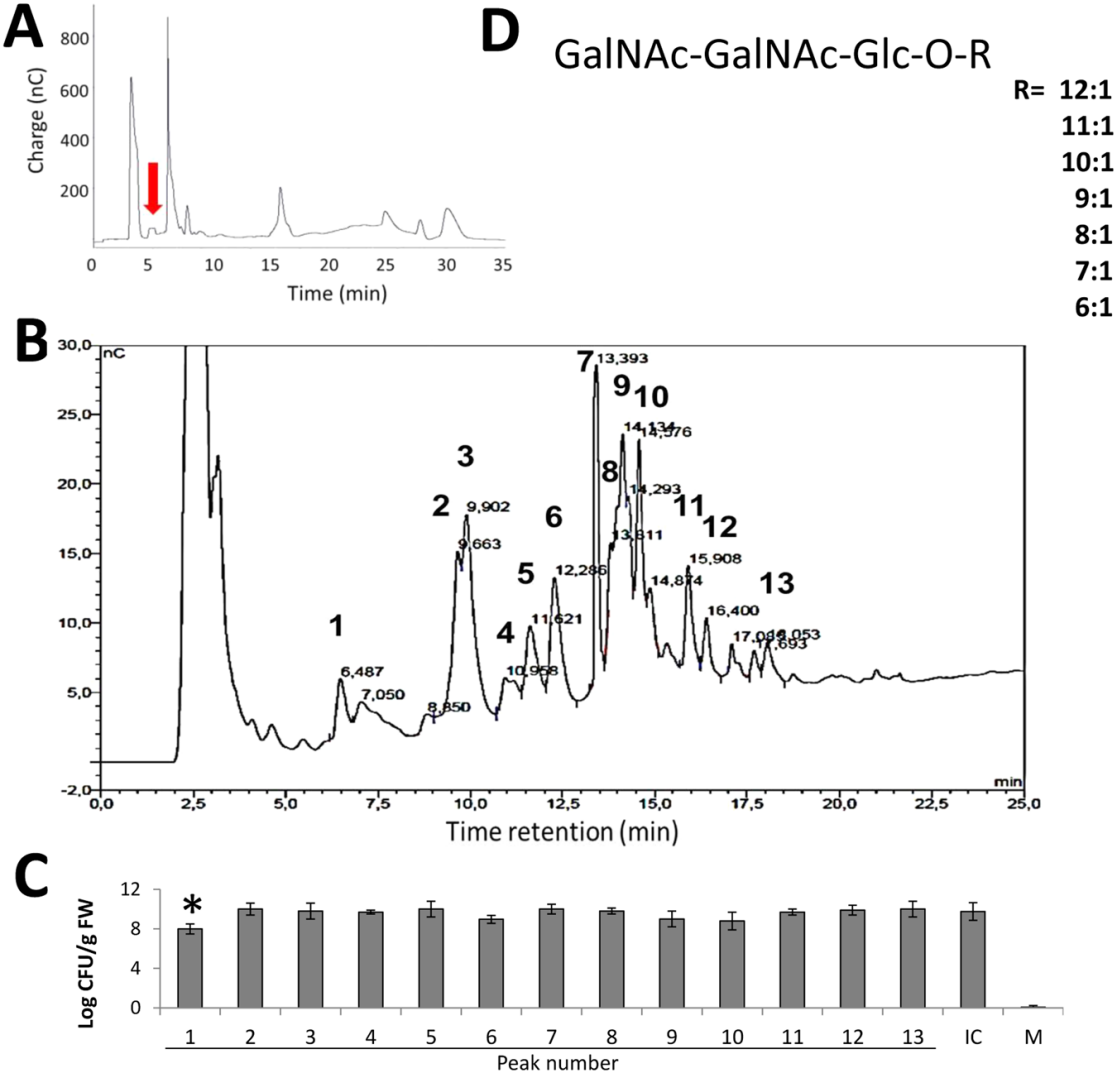
Purification and chemical structure of SAGs. (**A**) SAGs were purified from strawberry leaves by bioassay-guided normal phase (Zorbax-NH_2_) chromatography by collecting the unique fraction with plant defense inducing activity (arrow) which was later fractioned by (**B**) Carbopac PA-1 anionic exchange phase chromatography. (**C**) Bacterial count in leaves of Arabidopsis plants treated with each numbered peak and subsequently inoculated with the pathogen *Pseudomonas viridiflava* was determined and expressed as logarithm of colony forming units per leaf fresh weight (log CFU/g FW). Water-treated and thereafter inoculated plants are shown as infection control (IC) and non-inoculated plants as Mock treatment (M). (*) Denote statistically significant value using the DGC test (*p* < 0.05). Experiments were repeated four times with eight plants assayed for each peak. (**D**) Peak eluted at 6.487 minutes was chemically identified as the trisaccharide (GalNAc-GalNAc-Glc), esterified to a mono-unsaturated 6–12 carbons length fatty acid (R).

Aqueous extracts from strawberry leaves were fractionated by normal phase (Fig. 1A) and anionic exchange phase chromatography (Fig. 1B) and selected fractions were tested for protective effect against a pathogenic strain of *Pseudomonas viridiflava* on Arabidopsis plants (Fig. 1C). Leaves from pathogen-inoculated plants treated with the peak eluted at 6.487 minutes showed the greatest reduction of pathogenic bacteria, 1.75 logarithmic units, with respect to non-treated control plants, and was therefore selected for further studies and chemical identification as shown in Fig. 1D. The other fractions tested did not significantly reduce bacterial growth on inoculated plants. MALDI-TOF mass spectrum analysis of the selected fraction (Supplementary Figure S1) showed signals characteristic of an acylated trisaccharide with fatty acids of varying carbon chain length. Monosaccharides of this biologically active fraction were released by hydrolysis and identified as D-galactosamine and D-glucose in a 2:1 ratio (Supplementary Figure S2). In addition, fatty acids of the bioactive compound were derivatized and analyzed by gas-chromatography, which led to the identification of mono-unsaturated fatty acids, with a carbon chain ranging from 6 to 12 atoms. These analyses suggested a structure corresponding to a fatty acid glycoside composed of two molecules of N-acetylgalactosamine and a single glucose unit which in turn is bound by an ester linkage to the mono-unsaturated fatty acid (Fig. 1D). To the best of our knowledge this molecule has not been described previously, although similar fatty acid glycosides produced by microorganisms and plants have been reported^16^. Antibacterial and antifungal activities have been described for this group of compounds, and many acyl glycosides have been shown to act as emulsifier agents reducing both surface and interphase tension in organic/water liquid mixture. The latter property has been exploited for different applications in the oil, food, cosmetic, and pharmaceutical industries^17,18^. Although the chemical structure of SAGs indicates that these molecules may present amphipathic behavior, further studies have to be conducted to verify their possible surfactant characteristics.

### SAGs application activates innate plant defense systems and gives disease protection

**Figure 2:**
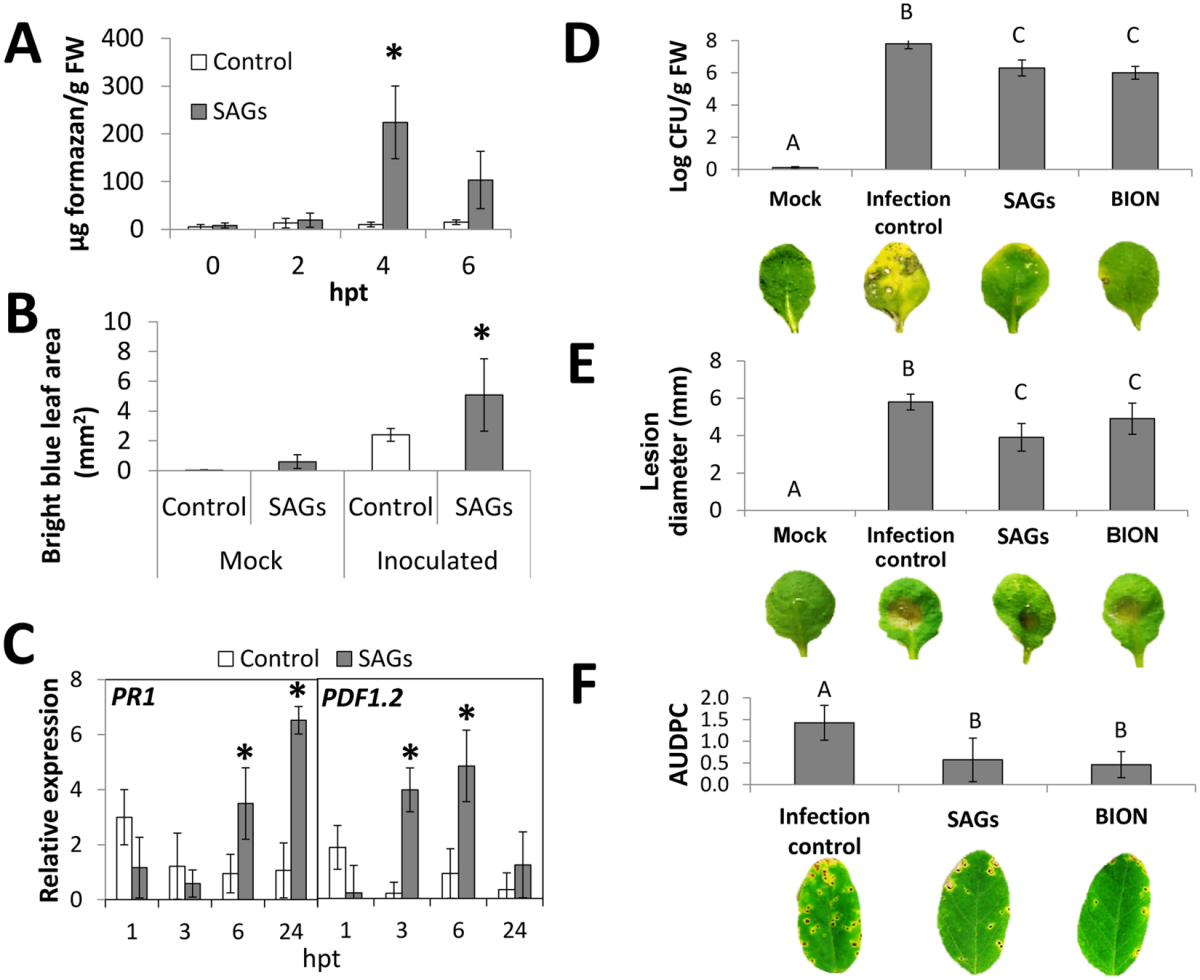
Pathogen defense-inducing activity of SAGs. (**A**) Superoxide radical production in leaves of plants after 0, 2, 4 and 6 hours post-treatment with SAGs (10 µg/ml) or water (control) measured by quantification of formazan produced after NBT histochemical staining. (**B**) Aniline blue staining to detect callose accumulation in leaves of SAGs- (10 µg/ml) or water-treated (control) plants later inoculated or not inoculated (Mock) with *Pseudomonas viridiflava*. Bright blue leaf areas (mm^2^) were measured from digital images of the stained leaves. Four leaves per plant and four plants per treatment were evaluated in three independent experiments for NBT and Aniline blue staining. (*) Denote values statistically different using the DGC test (*p* < 0.05). (**C**) Relative expression of *PR1* and *PDF1.2* genes calculated as 2^∆CT^ in water-treated (control) and SAGs-treated (10 µg/ml) Arabidopsis plants at 1, 3, 6 and 24 hours post treatment (hpt). (*) Denote statistically significant differences in RNA expression between control and SAGs-treated plants using the ∆CT method. Three technical and three biological replicates were analyzed for each qPCR reaction. (**D**) Protection assay on Arabidopsis plants inoculated with *P. viridiflava*. Bacterial population in plants pretreated with SAGs (10 µg/ml), BION 500 or water (infection control) were expressed as the logarithm of colony forming units per leaf fresh weight (log CFU/g FW). (**E**) Protection assay on Arabidopsis plants inoculated with *Botrytis cinerea*. Disease severity was measured as average of leaf lesion diameters in plants pretreated with SAGs (10 µg/ml), BION 500, or water (infection control). Non-inoculated plants were denoted as Mock. (**F**) Protection assay on soybean plants inoculated with *Corynespora cassiicola*. The area under the disease progress curve (AUDPC) 4, 7 and 10 days post pathogen inoculation was determined in soybean plants pre-treated with SAGs (100 µg/ml), BION 500 or water (infection control). Eight and six plants per treatment were used in the protection assays on Arabidopsis and soybean, respectively. Both experiments were repeated four times. Different letters indicate significant difference among treatments using the DGC test (*p* < 0.05). Images of symptoms in leaves from plants corresponding to each treatment are shown under each graph bar.

To study in more detail the protective effect of SAGs against plant pathogens, defense response mechanisms in SAGs-treated Arabidopsis plants were investigated. The activation of an innate immunity response in SAGs-treated plants was supported by an early and transient accumulation of reactive oxygen species (ROS) that reached a maximum 4 hours post treatment, as shown by spectrophotometrically quantifying purple formazan deposits in nitro blue tetrazolium-stained Arabidopsis leaves (Fig. 2A and Supplementary Figure S3). In addition, a minor callose deposition was observed in SAGs-treated plants after mock-treatment as a slight increase of brilliant areas in aniline blue-stained leaves (Fig. 2B and Supplementary Figure S4A). Callose deposition was enhanced in both SAGs-treated and water-treated plants after *P. viridiflava* infection (Supplementary Figure S4), although this increase was significant only in plants previously treated with SAGs (Fig. 2B). This result suggested that SAGs-treatment may have a priming effect on plant innate defense^32^.

Induction of defense-related genes in Arabidopsis was also associated with SAGs-treatment as expression levels of *PR1* gene, a genetic marker for the salicylic acid defense pathway, significantly increased at 6 and 24 hours post treatment. Similarly, expression of *PDF1.2* gene, a genetic marker for ethylene/jasmonate defense signaling, was up-regulated upon SAGs treatment (Fig. 2C). These results indicated that the SAGs-induced defense in Arabidopsis involves more than one defense signaling pathway and thereby may confer protection against different types of pathogens; an observation subsequently confirmed when SAGs-treated Arabidopsis plants showed increased disease protection against both a bacterial and a fungal pathogen. Deciphering the mechanism of action of a bioactive compound is very helpful in the development of a biostimulant or biocontroller, as such information will be of importance when optimizing use and time of application in any given agricultural system.

Arabidopsis plants spray-treated with SAGs 4 days before inoculation with a pathogenic strain of *P*. *viridiflava* resulted in an evident reduction of disease symptoms and 50-fold reduction of pathogenic bacterial population in infected leaves (Fig. 2D). This reduction is comparable to plants treated with the commercial biocontroller, BION 500 (Syngenta, Switzerland), based on the salicylic acid analog acibenzolar-S-methyl. A significant protective effect was also observed in Arabidopsis plants previously treated with SAGs when inoculated with the necrotrophic fungi *Botrytis cinerea* (Fig. 2E).

To evaluate the protective effect of SAGs in other plant species, soybean plants were sprayed with SAGs and three days later inoculated with the fungal pathogen *Corynespora cassiicola*, causal agent of the late season disease target spot. Soybean plants treated with SAGs at the V3 stage exhibited a 60% reduction in disease severity (Fig. 2F). These results indicated that SAGs may be used as biologically active ingredients in a biocontrol product designed for disease control in soybean.

### Antimicrobial effect and mechanism of action of SAGs on phytopathogens

**Figure 3:**
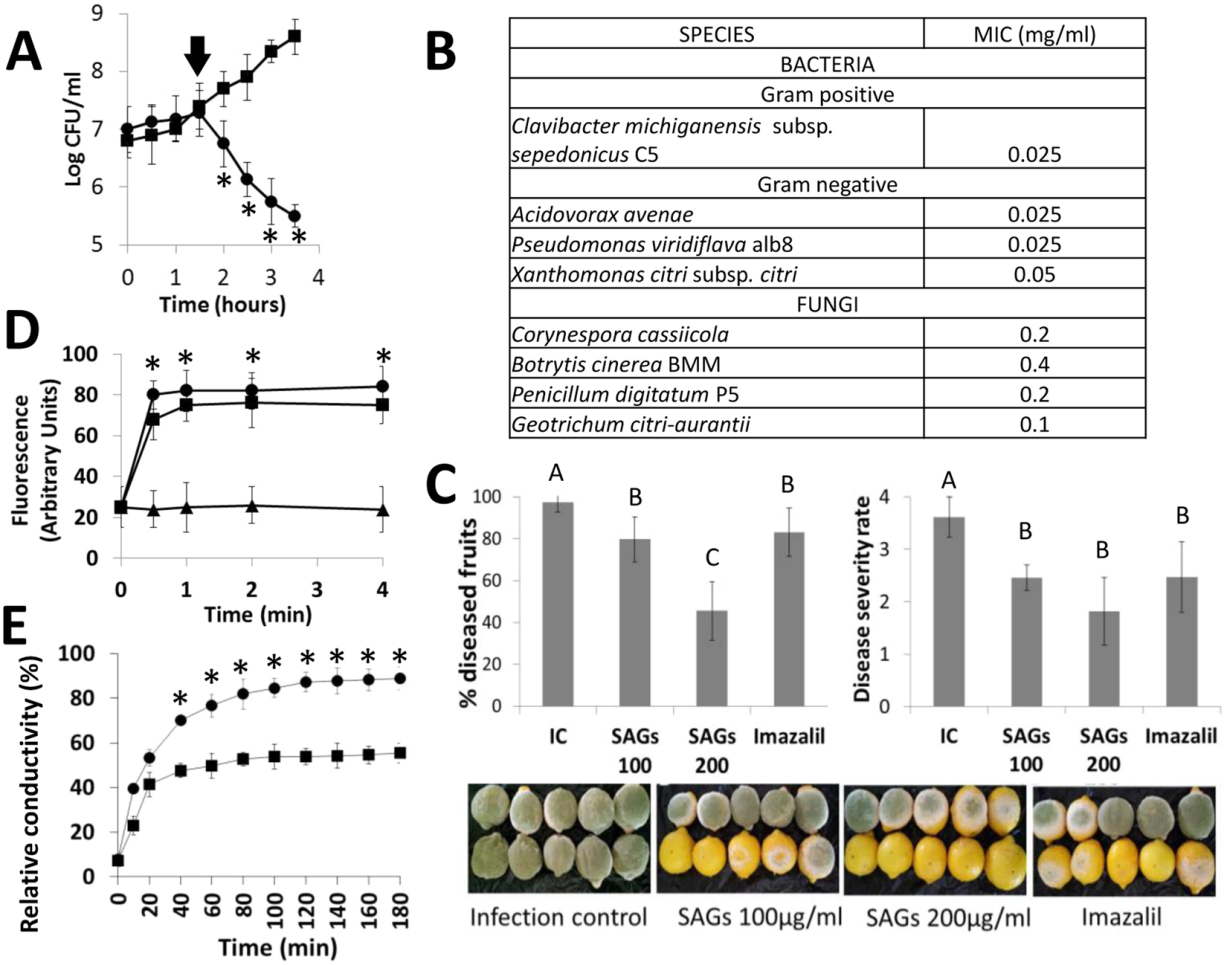
Antimicrobial activity of SAGs. (**A**) Effect of SAGs on *Clavibacter michiganensis* viability expressed as the logarithm of colony forming units per ml (log CFU/ml) at different time-points. SAGs application (arrow) at a final concentration of 60 μg/ml on fresh *C. michiganensis* liquid culture (circles) in comparison to non-treated bacteria (squares) was shown. (**B**) Antimicrobial activity of SAGs determined as Minimal Inhibitory Concentration (MIC) *in vitro* against different bacterial and fungal phytopathogens evaluated in at least three independent experiments. (**C**) Effect of SAGs-treatment on lemon fruits inoculated with *Penicillium digitatum*. Disease incidence (% diseased fruits) and disease severity (fruit area affected by fungus) were evaluated in inoculated fruits either treated with water (IC), SAGs at inhibitory (200 μg/ml) and sub-inhibitory (100 μg/ml) concentrations of pathogen growth, and 500 ppm of commercial fungicide Imazalil, 10 fruits were tested for each treatment in six independent assays. Different letters indicate significant difference among treatments using the DGC test (*p* < 0.05). Pictures in the lower panel show disease symptoms on lemon fruits for each treatment. (**D**) Effect on membrane permeability of bacterial cells treated with 60 μg/ml SAGs (squares), Valinomycin (circles) and water (triangles) at time-points indicated, using the intracellular fluorescent diSC3^5^. (**E**) Effect of SAGs (200 μg/ml) on membrane permeability of fungal mycelia cells at time-points indicated (circles) compared to water-treated cells (squares). Membrane leakage was determined by measuring relative conductivity of *C. cassiicola* mycelia. Results represent the average ± standard deviation of three independent experiments. (*) Denote statistically significant differences with control treatment.

As antimicrobial activity has been reported for many different glycolipids including fatty acyl glucosides^16,33,34^, *in vitro* growth inhibition studies against various plant bacterial and fungal pathogens were carried out with the purified SAGs presented in this study. A six-fold higher concentration of SAGs (60 μg/ml) than what was used in the purification protocol (10 μg/ml), was found to inhibit both bacterial cell growth and significantly decrease the number of bacteria in an actively growing culture of the tomato pathogen *Clavibacter michiganensis* (Fig. 3A). Further studies confirmed an antimicrobial effect against a wide spectrum of both bacterial and fungal phytopathogens (Fig. 3B). SAGs were found to prevent bacterial growth at about a ten-fold lower concentration as compared to the concentration needed to inhibit the fungal pathogens tested. The broad-spectrum of antimicrobial activity at low concentrations exhibited by SAGs could be of interest for applications in the food, cosmetic and pharmaceutical industry.

Antifungal activity of SAGs was also tested against the fungus *P*. *digitatum*, responsible for post-harvest decay in citrus fruits (Fig. 3C). Protection of *P. digitatum* infected lemon fruits with SAGs (100 μg/ml) was comparable to that obtained with Imazalil (Fungaflor 500EC, Janssen Lab., Belgium), a toxic fungicide commonly used in citrus production, whereas a higher concentration (200 μg/ml) clearly outperformed the commercial pesticide, significantly reducing the incidence of fungal disease symptoms in lemon fruits (Fig. 3C). SAGs and Imazalil were applied 6 hours after inoculation in order to imitate what occurs in the lemon packaging industry, where fruits suffer mechanical peel damages (primary infection sites) during harvest and transport to the packing plant where they later receive antifungal treatments. To study the possible underlying mechanism of the demonstrated antimicrobial activity of SAGs, taking into account the amphiphilic structure of these compounds, we applied the fluorescent dye diSC3^5^, which is sensible to dissipation of membrane potential and a good indicator of cellular membrane damage, to *C. michiganensis* cells later treated with SAGs. Addition of 60 μg/ml SAGs to diSC3^5^ containing bacterial cells induced a rapid increase of fluorescence very similar to that obtained with valinomycin, a well-known cell membrane potential dissipator (Fig. 3D). In addition, changes of the potential and/or permeability of the plasmatic membrane, seen as augmented fluorescence when increasing SAGs concentrations were successively tested, demonstrated a dose-dependent effect (Supplementary Figure S5). Similarly, action of SAGs on fungal membrane stability by measuring ion leakage, was studied. Relative ion-conductivity of *C. cassiicola* mycelia over time was significantly higher after SAGs treatment (200 μg/ml) as compared to mycelia treated with water (Fig. 3E). Cell membrane permeability of *C*. *cassiicola* mycelia treated with SAGs was noticeable higher (89%) when compared to water-treated control mycelia (55%) after 180 min of treatment. These results indicated that the antimicrobial effect observed for SAGs, at least partly, depends on membrane destabilization for both bacteria and fungus. A similar mechanism for antimicrobial activity has been suggested for closely related glycolipids including rhamnolipids^35^ and sophorolipids^36^. It is very probable that SAGs have amphiphilic properties that interact with both lipophilic and hydrophilic components, like many other saponin compounds, which often have antimicrobial activities and other biological effects due to their ability to complex with sterols in fungal membranes causing the loss of the membrane integrity with the formation of transmembrane pores^8^.

### SAGs as plant growth stimulator

**Figure 4:**
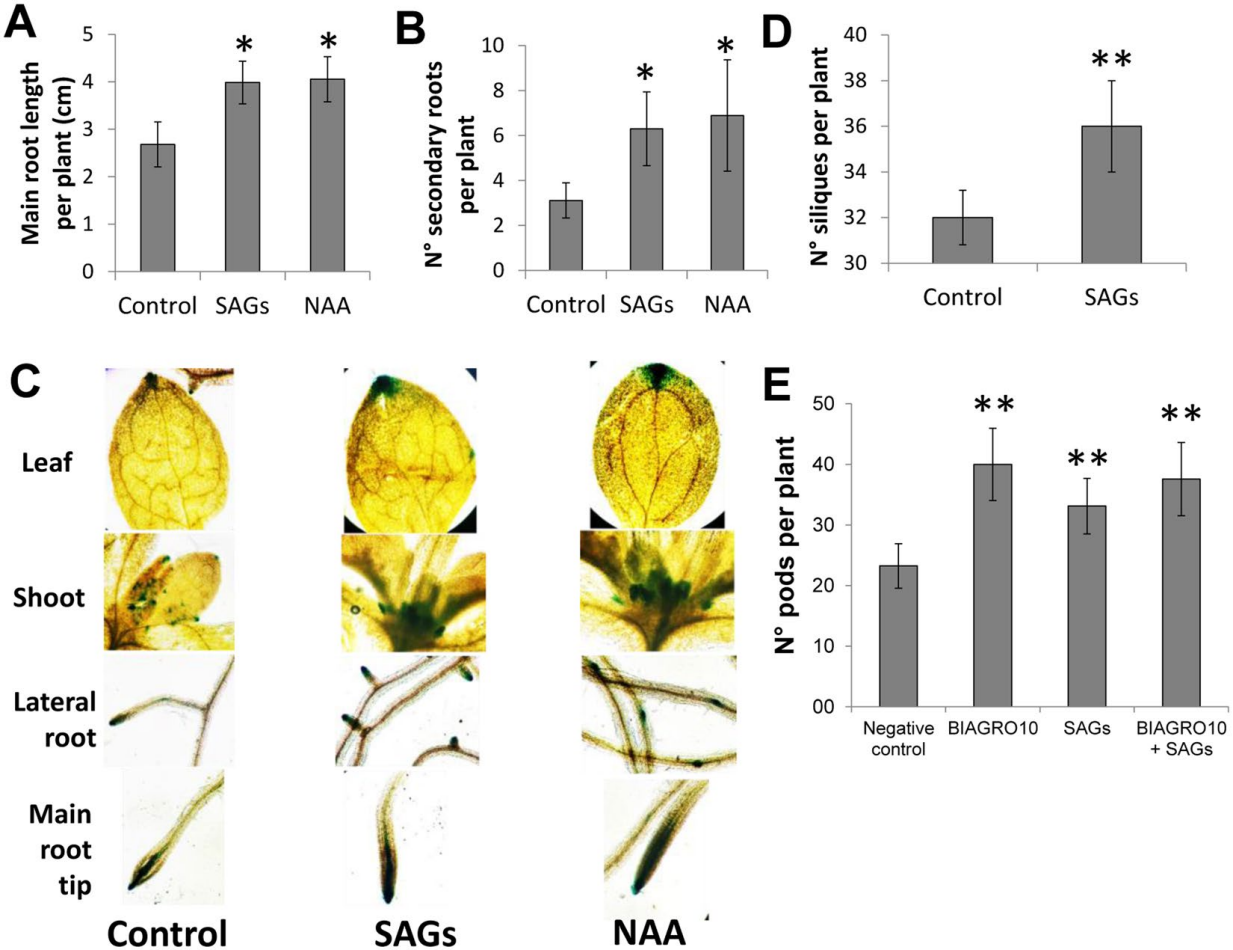
Plant growth stimulation of SAGs. (**A**) Main root length and (**B**) number of secondary roots of Arabidopsis plants grown on MS medium supplemented with SAGs (0.16 µg/ml) or naphthalene acetic acid (NAA), and control plants grown on non-supplemented medium was shown. Ten individual plants were analyzed per treatment, and the experiment was carried out three times. (**C**) Auxin-dependent *DR5::GUS* gene expression visualized as blue staining in Arabidopsis seedlings grown on SAGs (0.16 µg/ml) - or NAA-supplemented medium or on non-supplemented medium (control). Three independent experiments were performed with 10 plants per treatment. (**D**) Number of siliques of Arabidopsis plants, 5 weeks post-foliar treatment with SAGs (1.6 µg/ml) or water (Control). Three independent experiments with four random blocks of 6 plants per block were evaluated. (**E**) Effect of SAGs (50 µg/ml), BIAGRO10, SAGs + BIAGRO10, or water treatment (negative control) on number of pods in greenhouse-grown soybean plants. BIAGRO10 is a commercial bioinoculant based on the nitrogen fixing nodule-forming strain E109 of *Bradyrhizobium japonicum*. Four random blocks with 5 plants in each block were used in two independent experiments. Asterisks indicate statistically significant differences using DGC test (*p < 0.001, **p < 0.05).

An interesting observation was made in strawberry plants treated with SAGs-containing extracts during field trials conditions, as a visible increase in plant biomass where registered, indicating a possible plant growth stimulating effect of SAGs (data not shown). Another growth effect was detected in soybean, where an increase in seed pods per plants were observed when pathogen defense experiments were performed under controlled growth conditions in the greenhouse. To further study a possible growth and development stimulating effect of SAGs in plants, *in vitro* growth assays using Arabidopsis seedlings were conducted and, as shown in Fig. 4A–C and Supplementary Figure S6, the bioactive compounds (0.16 μg/ml) promoted both elongation of the primary root as well as development of lateral roots. It is important to notice that the plant growth stimulation of SAGs in Arabidopsis was performed at very low concentrations 0.16 µg/ml for *in vitro* experiments and 1.6 µg/ml for greenhouse-grown plants. The reason for this was due to a phytotoxic effect seen in Arabidopsis when higher concentrations of SAGs were applied.

It is well known that auxins are involved in lateral root emergence and development in plants^37–39^ and a similar result, as seen for SAGs-treated plants, on radicular growth was observed in Arabidopsis plants treated with the auxin NAA (5 ng/ml), although lateral root formation was less pronounced. Furthermore, when the effect of SAGs were tested in the Arabidopsis transgenic line DR5::GUS, in which auxin-responsive tissues are blue-stained when treated with the substrate 5-bromo-4-chloro-3-indolyl glucuronide (XGluc), seedlings grown on medium supplemented with SAGs (0.16 μg/ml) or NAA (5 ng/ml) showed an almost identical staining pattern in leaf tips, shoots, in tips and primordia of lateral roots and in the main root tip (Fig. 4C). In contrast, in non-treated Arabidopsis seedlings a much less pronounced blue staining of the same tissues was observed (Fig. 4C). This minor blue-staining in non-treated plants is most probably due to endogenous auxin effects. These results suggested that the observed plant growth stimulation by SAGs could partly depend on the auxin response pathway by a direct activation of auxin signal transduction pathways or by an increase of endogenous auxins levels, but further studies are needed before a direct relationship with auxin signaling can be concluded. Additionally, foliar application of a higher concentration of SAGs (1.6 µg/ml) on mature Arabidopsis plants produced a significant increase (12%) of siliques per plant (Fig. 4D). Likewise, soybean plants sprayed with a SAGs solution (50 μg/ml) developed over 40% more seed pods per plant than water-treated control plants (Fig. 4E).

## Conclusions

The different effects exerted by SAGs on plants and fruits showed a concentration dependent response. At higher concentrations (>100 µg/ml) SAGs exert a direct antimicrobial effect on phytopathogens, most likely by damaging the microbial plasma membrane. However, such high concentrations are phytotoxic for some plant species (i.e. Arabidopsis). Therefore application of these concentrations could be exploited to manage post-harvest fruit diseases before (preventive) or after (curative) infection occurs. At lower concentrations, (<50 µg/ml) where no antimicrobial or phytotoxic effect is observed, SAGs could be employed as elicitors of plant defense for disease management. At these concentrations a growth stimulating effect was observed in soybean and it is therefore plausible that application of concentrations in this range could have a dual effect, as activator of defense and stimulator of growth in this crop. However, before any final conclusions can be made field trials in different agroecosystems and crops will have to be performed.

## Methods

### Purification of SAGs

SAGs were obtained from freshly harvested *Fragaria x ananassa* leaves. Extraction was performed in distilled water acidified with 0.1% of trifluoroacetic acid (TFA) by homogenizing plant material (0.1 g fresh weight/ml of solvent) and shaking at 50 rpm (4 °C) for 24 hours. Clarified extract was recovered after centrifugation at 10.000 × g for 15 min and fractioned by preparative chromatography using a solid phase extraction (SPE) cartridge containing a C18-E matrix (Phenomenex, USA). Clarified extract was loaded on the column followed by washing with 0.1% TFA diluted in water. Initial loading volume together with washing flow-through was collected as a preliminary purification step as the SAGs molecules were not absorbed by the column matrix.

Recovered fraction was 40 times-concentrated in a vacuum concentrator (SpeedVac, Thermo Scientific), and further purified by HPLC using a reverse phase chromatography (SOURCE 5RPC, GE Healthcare Biosciences AB) column and a binary solvent system (distilled water and methanol, both acidified with 0.1% TFA). SAGs were recovered in the flow-through, which was 2 times vacuum-concentrated and purified using a normal-phase HPLC Zorbax-Amino (Waters, USA) column with an isocratic gradient of 80% acetonitrile (1 ml/min). The desired fraction was collected at around 5 minutes, vacuum-concentrated to dryness and resuspended in 1 ml of distilled water. In a final purification step a High Performance Anionic Exchange Chromatography with Pulse Amperometric Detector (HPAEC-PAD) system was used with a Carbopac PA-1 (Dionex, USA) column where bound SAGs were eluted by a gradient of a tripartite solvent (200 mM NaOH as solvent A, water as solvent B and 1 M AcONa as solvent C). An initial solvent gradient of 25% A, 75% B and 0% C was maintained for 10 min, followed by a linear gradient where concentrations of solvents B and C were changed until reaching a final solution after 30 min containing 25% A, 25% B and 50% C(1 ml/min) where SAGs were recovered at 6.49 minutes.

### Determination of SAGs chemical structure

First matrix-assisted laser desorption/ionization-time-of-flight (MALDI-TOF) mass spectrometer analysis was performed. Pure SAGs were neutralized using acetic acid, vacuum-concentrated to dryness and analyzed by MALDI-TOF mass spectrometry using a 2,5-dihydroxybenzoic acid (DHB) matrix in reflectron positive mode. Monosaccharide composition was determined by dissolving SAGs in distilled water and later hydrolyzing in 2 N trifluoroacetic acid (TFA) during 4 hours at 100°C. The hydrolyzed sample was again vacuum-concentrated to dryness, dissolved in distilled water and analyzed by HPLC to identify released monosaccharides by comparison with standard samples of D-galactosamine, D-glucosamine, L-fucose, D-manose, D-galactose and D-glucose. Analysis was made by HPAEC-PAC using an anionic-exchange Carbopac P20 (Dionex, USA) column and a flow rate of 0.5 ml per minute. Neutral and amino monosaccharides were eluted by an isocratic program of 6% of solvent A (200 mM NaOH) and 94% of solvent B (distilled water). Acidic monosaccharides were eluted by a tripartite solvent (200 mM NaOH as solvent A, water as solvent B and 1 M AcONa as solvent C) using a isocratic program of 24% A, 62% B and 14% C.

Fatty acids composition was determined by gas chromatography of methyl ester derivatives. SAGs were hydrolyzed in 1.67% NaOH at room temperature with gentle shaking for 24 h and thereafter acidified with 0.5 M HCl and three times-extracted with an equal volume of dichloromethane. Organic phases were recovered, pooled and concentrated to dryness. The resulting residue was dissolved in 0.5 ml toluene and an equal volume of 20% boron trifluoride in methanol was added and incubated at 80 °C during 1 h under an atmosphere of nitrogen. After cooling to room temperature, the sample was washed 3 times by adding an equal volume of distilled water and recovering the organic phase, which was 3 times-extracted with toluene. Fatty acid analysis was performed by gas chromatography on a capillary column (Ultra 1,25 m × 0.20 mm) with an initial temperature of 80 °C for 2 min followed by a temperature-increase up to 290 °C (10 °C /min) during a total time of 30 min.

### Disease resistance assay in Arabidopsis

Healthy 4-week-old Arabidopsis Col-0 plants sprayed with 10 μg/ml SAGs were 4 days later inoculated with the bacterial pathogen *Pseudomonas viridiflava* alb8^40^ or the fungal pathogen *Botrytis cinerea* BMM^41^. Treatment with 0.4 mg/ml of the commercial biocontrol product BION 500 (Syngenta, Switzerland) was included as a plant defense-induction control whereas plants sprayed with sterile distilled water were used as negative controls and water-treated inoculated plants as infection controls. *Pseudomonas viridiflava*-inoculated plants were subsequently incubated in plant culture chambers under controlled growing conditions (26°C, 80% RH, 16 h photoperiod) and four days post bacterial inoculation leaf disease symptoms were recorded and colony forming units per grams of fresh weight determined by homogenization of leaves from infected plant and plating serial dilutions on LB-plates.

In addition, *B. cinerea*-inoculated plants were incubated in culture chambers at 22°C, 80% RH in darkness and three days later foliar lesion diameters were measured and recorded. Concentrations of SAGs used in the experiment were sub-inhibitory to both bacterial and fungal pathogen growth. Eight plants per treatment were assayed in four independent experiments.

### Detection of reactive oxygen species (ROS) in Arabidopsis leaves

*In situ* detection of superoxide radical production in leaves of Arabidopsis was carried out by NBT histochemical staining^42^, with minor modifications. Plants were subjected to 10 μg/ml SAGs- or distilled water-treatments by foliar spraying. At 0, 2, 4 and 6 hours post treatment leaves were detached from treated plants, fresh weight determined and immersed in a 50 mM potassium phosphate buffer (pH 7.8) containing 0.1% NBT and 10 mM sodium azide. Stained leaves were vacuum-infiltrated by two vacuum shock-treatments for 1 min at 100 mm Hg, and thereafter incubated for 1 hour in darkness (without vacuum). After dark-incubation, leaves were first immersed in 96% (v/v) ethanol, to eliminate remaining chlorophyll, and then clarified and conserved in a solution of lactic acid/glycerol/water (3:3:4 v/v/v). Superoxide production was visualized as purple formazan deposits within leaflet tissues (Supplementary Figure S3), which were extracted and solubilized to spectrophotometrically quantify the superoxide accumulation in leaves, following the method of Grellet-Bournonville and Diaz-Ricci^43^. Briefly, NBT-stained and clarified leaves were homogenized in a mix of KOH (2 N):chloroform (1:1), the organic phase was recovered, vacuum-concentrated to dryness and the formazan extracted was dissolved in 350 µl dimethylsulfoxide and 300 µl KOH 2 N. Formazan was quantified in a spectrophotometer at 630 nm and expressed as µg of formazan per gram of leaf fresh weight using a calibration curve. Four leaves per plant were collected and four plants per treatment were evaluated in three independent experiments.

### Callose accumulation in Arabidopsis leaves

Callose deposition was visualized using aniline blue dye^44^. Detached leaves of treated Arabidopsis Col-0 plants were discolored in 96% ethanol, and gradually rehydrated by being submerged sequentially in 50% ethanol, 25% ethanol and finally in 67 mM K_2_HPO_4_ (pH 12). After rehydration, leaves were stained for 1 hour with 0.05% aniline blue in darkness, and finally immersed in 30% glycerol before being analyzed under UV-light in a fluorescence microscope, to visualize callose accumulation as bright blue areas (Supplementary Figure 4 A). Before staining, Arabidopsis plants were sprayed with either 10 μg/ml SAGs or distilled water, and 6 days post-treatment detached leaves were stained as described above. In addition, a group of treated plants were inoculated with *P. viridiflava* alb8 4 days after SAGs- and water-treatment and stained with aniline 2 days later. Four leaves per plant were collected and four plants per treatment were evaluated in three independent experiments. Quantification of callose deposition was measured from digital images obtained from microscopic visualizations which correspond to leaf areas of 25 mm^2^, using the ImageJ (Image Processing and Analysis in Java) software (NIH, USA). Briefly, each image was converted to greyscale mode, a line with known length was drawn to define the real scale based on the eyepiece micrometer of the microscope. For all images threshold values for bright blue area was defined by only considering values ranging from 50–255 in the histograms showed. Selected areas were thereafter converted into red stained areas by the software (Supplementary Figure 4B) and measurement of the detected area was calculated using the analyze particle option, setting minimum particle size as 0.00002 cm^2^. Automatically, Image J software generated a new image with the detected areas (Supplementary Figure 4 C) and a results table obtained to each image (Supplementary Figure 4D). The calculation of the average Calculated Total Area value (cm^2^) for each microphotography was performed for each treatment and graphed as the bright blue area (mm^2^) per 25 mm^2^ of total leaf area.

### Analysis of gene expression in Arabidopsis

Evaluation of gene expression in Arabidopsis plants was performed by real-time PCR. Total RNA was purified from leaves of Arabidopsis plants at 1, 3, 6 and 24 hours after foliar spraying with 10 μg/ml SAGs or distilled water. Detached leaves from treated plants were homogenized in liquid nitrogen using a mortar and pestle and total RNA was extracted by the Trizol method^45^. Briefly, 150 mg of leaves were homogenized in 1 ml of Trizol reagent, purified in a chloroform:isoamyl alcohol mix, and finally treated with DNAse I. Purity and quality of extracted RNA was determined by spectrophotometry and electrophoresis, respectively. Retrotranscription was performed with the reverse transcriptase M-MLV enzyme (Thermo Scientific) following the manufacturer´s instructions. Resulting cDNA was analyzed by real-time PCR using iQTM SYBR® Green Supermix (BioRad). Expression of *PR1* (At2g14610; forward primer: GTCTCCGCCGTGAACATGT; reverse primer: CGTGTTCGCAGCGTAGTTGT) and *PDF1.2* (At5g44420; forward primer: TTTGCTTCCATCATCACCCTTA; reverse primer: GCGTCGAAAGCAGCAAAGA) genes were studied. The housekeeping *EF1* gene (At1g18070; forward primer: AGCACGCTCTTCTTGCTTTC; reverse primer: GGGTTGTATCCGACCTTCTTC) was used as reference gene whose expression did not change at each time point and treatment studied. Gene expression levels were measured using the ∆C_T_ method calculated as 2^∆C^_T_ to water-treated (control) or SAGs-treated plants^46^. ∆C_T_ is the subtraction between reference gene C_T_ value and target gene (*PR1* or *PDF1.2*) C_T_ value. The least number of cycles at which enough amplified product accumulates to yield a detectable fluorescent signal is called the threshold cycle, or C_T_. Three technical and three biological replicates were analyzed for each treatment.

### Disease resistance assay in soybean

Healthy soybean (*Glycine max*) seeds of elite variety A8000 RG were sown in 4 l pots with soil, germinated in the greenhouse and grown to vegetative stage V3 (unifoliolate and first three trifoliolate leaves fully developed), when plants were sprayed with 100 μg/ml SAGs and three days later inoculated with virulent strain C4 of the pathogenic fungus *C. cassiicola*^47^, causal agent of soybean target spot. It should be noted that the concentration of SAGs used in the experiment were sub-inhibitory for growth of *C. cassiicola*. In addition, pre-treatment with sterile distilled water, or with 0.4 mg/ml of the commercial biocontrol product BION 500 were evaluated. Target spot-affected areas were evaluated on V3 and V4 plant segment leaves at 4, 7 and 10 days post-inoculation. Severity Index was calculated for each plant and each time point post-inoculation^47^ and the area under the disease progress curve (AUDPC) for each treatment was determined^48^. Four independent experiments were performed, and six soybean plants per treatment were used in each assay.

### Liquid medium growth and membrane depolarization assays in *Clavibacter michiganensis*

The antimicrobial activity of SAGs was evaluated against the bacterial strain *C. michiganensis* subsp. *sepedonicus* C5 growing in liquid LB medium at 120 rpm and 26°C with an initial OD_600_ adjusted to 0.1 (7 log colony forming unit per ml) and OD_600_ measurements taken every half-hour. SAGs were added to growing bacteria after 1.5 hours of growth to a final concentration of 60 μg/ml. Membrane depolarization of *C. michiganensis* was monitored as changes in fluorescence emission intensity of the membrane-potential-sensitive dye diSC3^5,49^. *C. michiganensis* was grown in LB liquid medium to mid log phase growth (OD_600_ = 0.5) where after cells were harvested by centrifugation and washed once (5 mM glucose and 20 mM HEPES pH 7.3) and resuspended to a concentration of OD_600_ of 0.05 in the same buffer. Aliquots of 100 µl were placed into quartz cuvettes containing 2.0 ml of 5 mM glucose, 100 mM KCl and 5 mM Na HEPES buffer pH 7.2. After addition of 0.4 µM diSC3^5^, cuvettes containing bacterial cells were incubated at 26°C until a stable reduction of fluorescence (around 5 min), indicating incorporation of the dye into the bacterial membrane, was achieved. SAGs (60 μg/ml), Valinomycin (1 μM plus 100 mM KCl) or water were thereafter added and the dye fluorescence increase was recorded at 622 nm (excitation wavelength) and 670 nm (emission wavelength) with a RF-5301PC spectrofluorometer (Shimadzu, Kyoto, Japan) at 0, 0.5, 1, 2 and 4 min. The antibiotic Valinomycin, a well-known K^+^-selective ionophore that changes the bacterial membrane potential, was used as positive membrane-destabilizing control. A SAGs dose-response assay on membrane permeability of *C. michiganensis* was performed measuring the fluorescence after 1 minute at different concentrations (0, 7, 15, 30, 60 and 90 μg/ml) of SAGs. Experiments were repeated at least three times under identical experimental conditions.

### Action on fungal cell membrane permeability

A method described by Elsherbiny and Taher^50^ was assayed. Briefly, *C*. *cassiicola* was cultured on Potato-Dextrose-Agar plates for 10 days and mycelial disks of 5 mm in diameter were taken from the margins of each plate to inoculate flasks with 100 ml of Sabouraud Maltose Broth. After 48 h of incubation with shaking at 150 rpm at 25°C, SAGs at a final concentration of 200 μg/ml was added. Flasks without application of SAGs were used as fungal control. After an additional 48 h of incubation under the same conditions, mycelia were collected from both treated and untreated flasks, washed twice with double distilled water and vacuum-filtrated. A measured amount of mycelia (0.5 g) was suspended in 20 ml of double distilled water and conductivity was measured after 0, 10, 20, 40, 60, 80, 100, 120, 140, 160 and 180 min with a pH/CON 510 series conductivity meter (Oakton, Illinois, USA). Finally, the mycelia was boiled for 5 min to measure total conductivity and the relative conductivity of mycelia was calculated (Relative conductivity (%) = conductivity/total conductivity × 100). The experiment was repeated three times.

### Antimicrobial assay on agar plate

Antimicrobial activity of SAGs was evaluated against several bacterial plant pathogen species^51^. SAGs were diluted in sterile distilled water ranging from 6.25 to 100 μg/ml, and a drop of 10 μl of each was placed on LB agar plate until absorption. Molten sterile soft agar (0.7% in water) were cooled to 40 °C and inoculated with 10^7^ cells/ml of an overnight culture of each bacterial phytopathogenic strain (*C. michiganensis* subsp. *sepedonicus* C5, *Acidovorax avenae*, *Pseudomonas viridiflava* alb8, *Xanthomonas citri* subsp. *citri*), poured on LB agar plates and incubated at 28°C. After 24 to 48 hours of incubation at 28°C, the zone of growth inhibition around each SAGs drop was observed. Minimal Inhibitory Concentration (MIC) was taken as the lowest concentration of SAGs that showed bacterial growth inhibition. LB medium was replaced with Cadmus medium to evaluate *Xanthomonas citri* subsp. *citri*.

Antifungal growth activity of SAGs was evaluated in a similar way as for bacteria. SAGs were diluted in sterile distilled water with a final concentration ranging from 5 to 1000 μg/ml, and a drop of 50 μl of each concentration was placed onto Potato-Dextrose-Agar (PDA) plates until absorption. Molten sterile soft agar (0.7% in water) was cooled to 40°C and inoculated with 10^4^ conidia/ml with an overnight culture of each of the following fungal phytopathogenic strains (*Corynespora cassiicola, Botrytis cinerea* BMM, *Penicillium digitatum* P5 and *Geotrichum citri-aurantii*), poured on PDA plates containing drops of different concentrations of SAGs and incubated at 28°C for 48 to 72 hours. After incubation zones of growth inhibition around each SAGs dilution was observed and MIC was determined for each fungal strain. Each phytopathogen was evaluated in at least three independent experiments.

### Assays of postharvest disease protection in lemon fruits

Lemon fruits previously disinfected by immersion in a 10% sodium hypochlorite solution and thereafter washed in distilled water, were inoculated by superficially wounding (1 mm in diameter and 2 mm in depth) the fruit surface by punching with a steel bar, and later apply a 20 μl aliquot of a 10^6^ spores/ml suspension of the virulent strain P5 of *P. digitatum*. Infected fruits were sprayed with two concentrations of SAGs, 100 and 200 μg/ml, 6 hours after pathogen inoculation. As a positive control of fungal disease protection a commercial fungicide, 500 ppm Imazalil (Fungaflor 500EC, Janssen Lab., Belgium) was applied 6 hours after inoculation, while distilled water-treated lemons were used as infection controls. All treated fruits were incubated in a growth chamber at 25°C and 90% RH. Six days post pathogen-inoculation, fruit inspection was performed and the incidence (% of fruits with symptoms) and severity of the disease (fruit surface affected by mycelial development) were evaluated. Numerical visual rating with values ranging from 0 (without development of mycelium) to 4 (fruit completely covered with mycelium) was conducted. From the evaluation of the surface affected by mycelium for each fruit, a severity rate was calculated as the average frequency of affected fruits for each treatment. Evaluations were performed in 10 fruits for each treatment, and in six independent assays.

### Growth promotion studies in Arabidopsis

Plants of Arabidopsis Col-0 and the DR5::GUS transgenic line were grown using a vertically oriented agar-plate culture system to evaluate aerial growth and root development^52^. SAGs (0.16ug/ml) were added to molten MS-medium (50°C) and poured onto agar plates. Phenotypes were generally observed at 7, 10 and 14 days after seed germination. For comparison, auxin effects were evaluated in plants grown in MS-medium supplemented with 5 ng/ml naphthalene acetic acid (NAA). Number of secondary roots per plant was determined at 10 days post germination using a light microscope. Main root length per plant was measured using the ImageJ software 1.48 v (NIH, USA) for digital imaging of roots. Experiments were repeated three times using 10 plants in each experiment. The DR5::GUS transgenic plants carries a strong synthetic auxin-responsive promoter (DR5) driving the β-glucuronidase reporter gene (*gus*A), which can be used to detect auxin distribution and responses in plant tissue by histochemical staining^53^. To visualize *gus*A expression in different plant tissues, seedlings grown as described previously were collected and submerged in the chromogenic substrate 5-bromo-4-chloro-3-indolyl glucuronide (X-Gluc) for 10 min at room temperature and then incubated at 37°C in the dark for 16 h. After dark-incubation seedlings were rinsed with 50 mM sodium phosphate buffer pH 7.0, cleared with 95% (v/v) ethanol and transferred to 70% (v/v) ethanol. Images of GUS-stained Arabidopsis tissue were recorded using a light microscope at 10X magnification equipped with a digital camera. Experiments were repeated three times using 10 plants for each experiment.

Plant growth promoting effects of SAGs on greenhouse-grown (25°C, 16 h photoperiod and 70% HR) Arabidopsis Col-0 plants were assayed by spraying SAGs (1.6 ug/ml) on aerial parts of healthy 3 weeks old plants in the beginning of flowering (onset of inflorescence). Total silique number per plant was evaluated 5 weeks after treatment. Plants sprayed with distilled water were used as control treatment. Experimental design consisted in 4 random blocks of 6 plants per block for each treatment; the experiment was repeated three times.

### Pod setting in soybean

*Glycine max* plants (A8000 RG, maturity group VIII), sown in pots with a sterile low-fertility soil substrate and grown under controlled greenhouse conditions, were treated by spraying aerial parts with SAGs (50 μg/ml) and water (negative control) together with a siliconized adjuvant, nonylphenolethoxylate (0.3%), at plant growth stages V1, V7, R1 and R3. Vegetative V1 and V3 stages correspond to plants with a unifoliate leaf, and plants with the unifoliate leaf and first three trifoliate fully developed leaves, respectively. Reproductive R1 and R3 stages correspond to soybean plants at onset of flowering, and at beginning of pod formation, respectively. As positive growth promotion control, treatment of soybean seeds with the commercial bioinoculant BIAGRO10 (BIAGRO S.A., Argentina), based on nodule-inducing strain *Bradyrhizobium japonicum* 109 was carried out according to instructions from the manufacturer (77 g/L for 190 kg of seeds). Experiments were carried out in a greenhouse with temperatures ranging from 25 to 30°C and a controlled photoperiod of 18 hours light (12000–18000 lux). For each treatment, 4 random blocks with 5 plants in each were used in two independent assays. Once plants had reached stage R8, total number of pods per plant was determined for each treatment and the results were statistically analyzed by ANOVA using a random block experimental design.

### Statistical analysis

Software InfoStat version 2018 (http://www.infostat.com.ar) was used for statistical analysis of all data and ANOVA by Di Rienzo, Guzmán and Casanoves (DGC) comparisons with *p*-value <0.05 was performed to detect statistically significant differences among treatments.

## Supplementary information

Supplementary Information.
