# Supplementary material for: Strawberry fatty acyl glycosides enhance disease protection, have antibiotic activity and stimulate plant growth
Source: Sci Rep. 2020 May 18;10:8196. doi: 10.1038/s41598-020-65125-7 (PMC7235083; doi:10.1038/s41598-020-65125-7)
Supplement: Supplementary file 1 — Supplementary Information. [file 41598_2020_65125_MOESM1_ESM.pdf]

Supplementary Information

Strawberry fatty acyl glycosides enhance disease protection, have antibiotic activity and stimulate plant growth

Grellet Bournonville Carlos[1a], Filippone María Paula[1a], Di Peto Pía de los Ángeles[1a], Trejo María Fernanda[1], Couto Alicia Susana[3], Mamaní de Marchese Alicia[2], Díaz Ricci Juan Carlos[4], Welin Björn[1*] and Castagnaro Atilio Pedro[1*]

Supplementary Figure S1

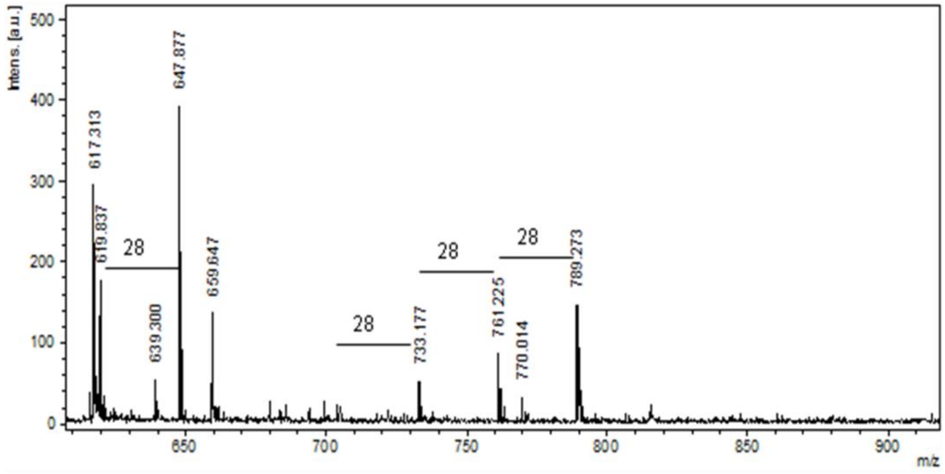

Supplementary Figure S1. Matrix assisted laser desorption/ionization (MALDI)-time-of-flight (TOF) mass spectrometer analysis. Pure SAGs were analyzed using a 2,5-dihydroxybenzoic acid (DHB) matrix in reflectron positive mode.

Supplementary Figure S2

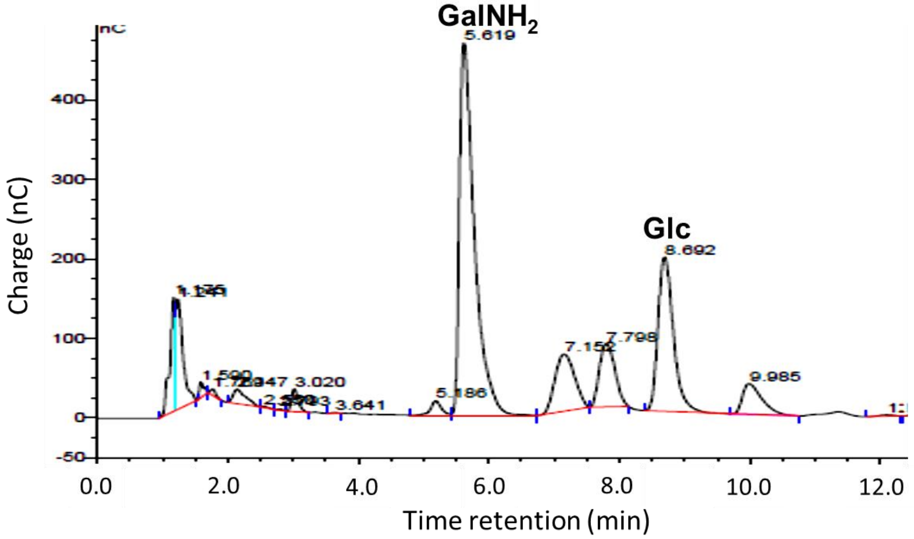

Supplementary Figure S2. Monosaccharide composition of SAGs. Neutral monosaccharides and amino sugars released by acid hydrolysis were identified by HPAEC-PAD. D-galactosamine (GalNH2) and D-glucose (Glc) in a 2:1 ratio were detected as the main monosaccharides present.

Supplementary Figure S3

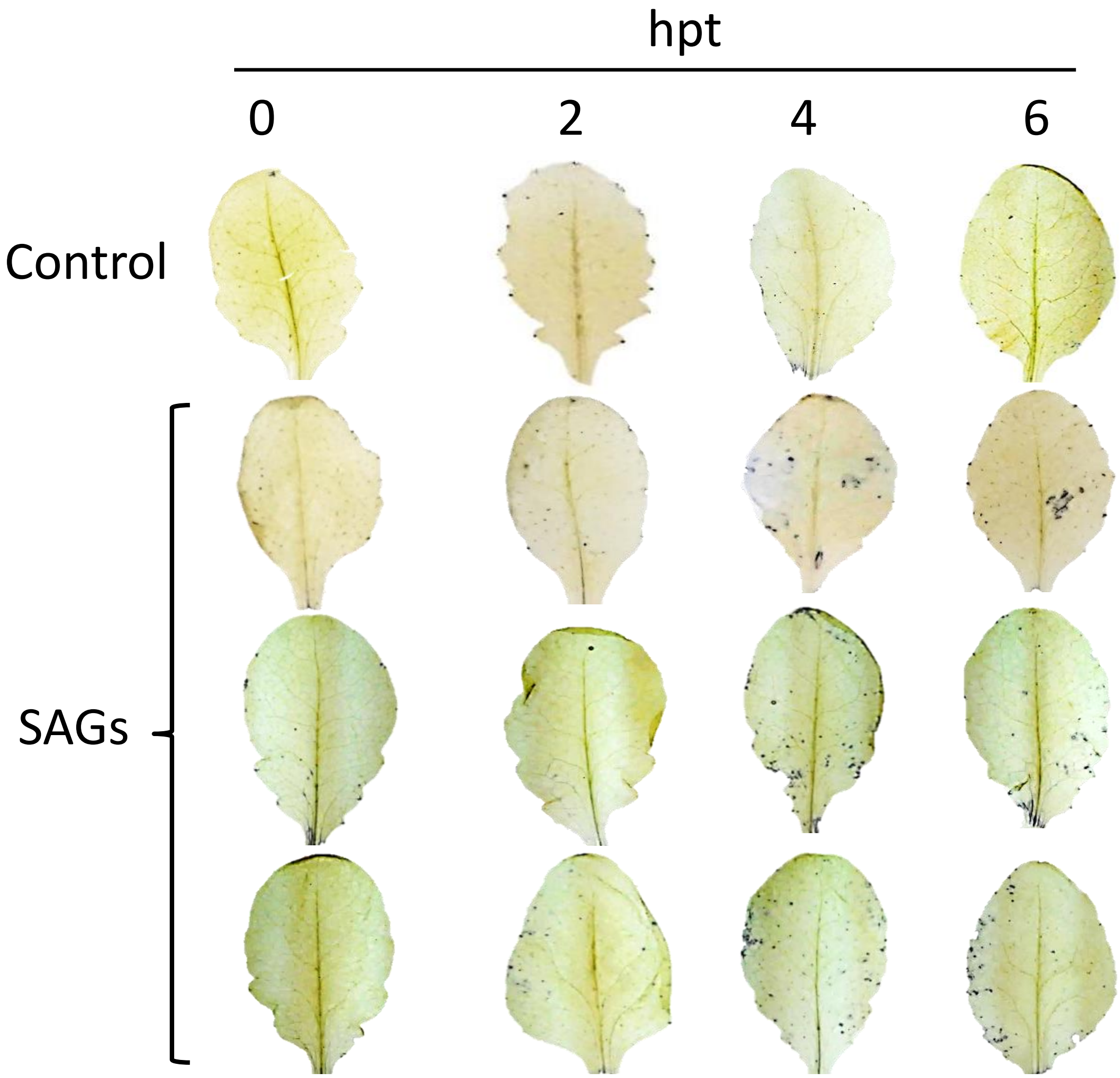

Supplementary Figure S3. NBT histochemical staining to visualize superoxide radical production in leaves of plants at 0, 2, 4 and 6 hours post-treatment (hpt) with SAGs (10ug/ml) or water (control). Purple deposits show superoxide radical accumulation. Images represent the results obtained from three independent experiments.

Supplementary Figure S4

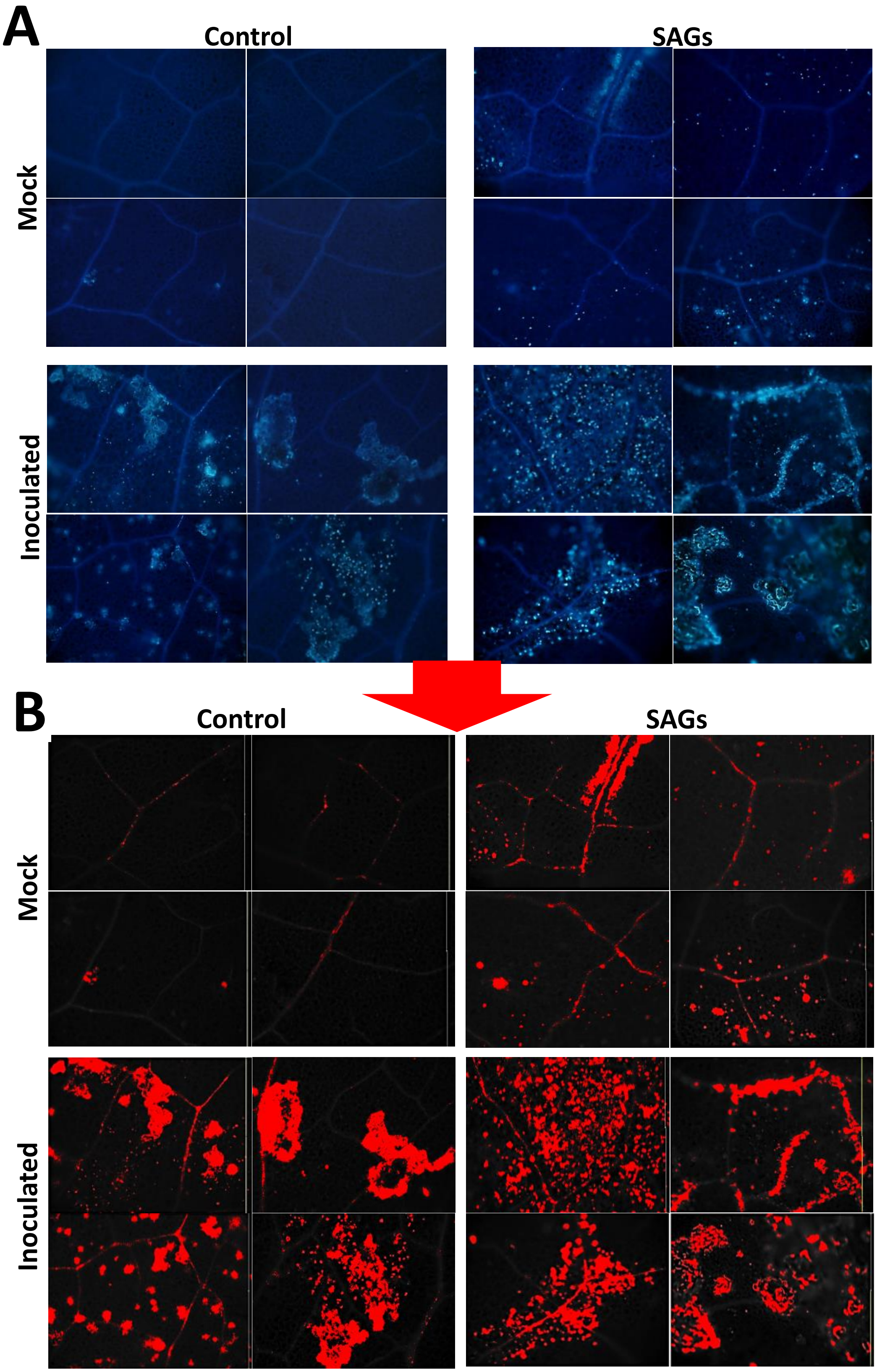

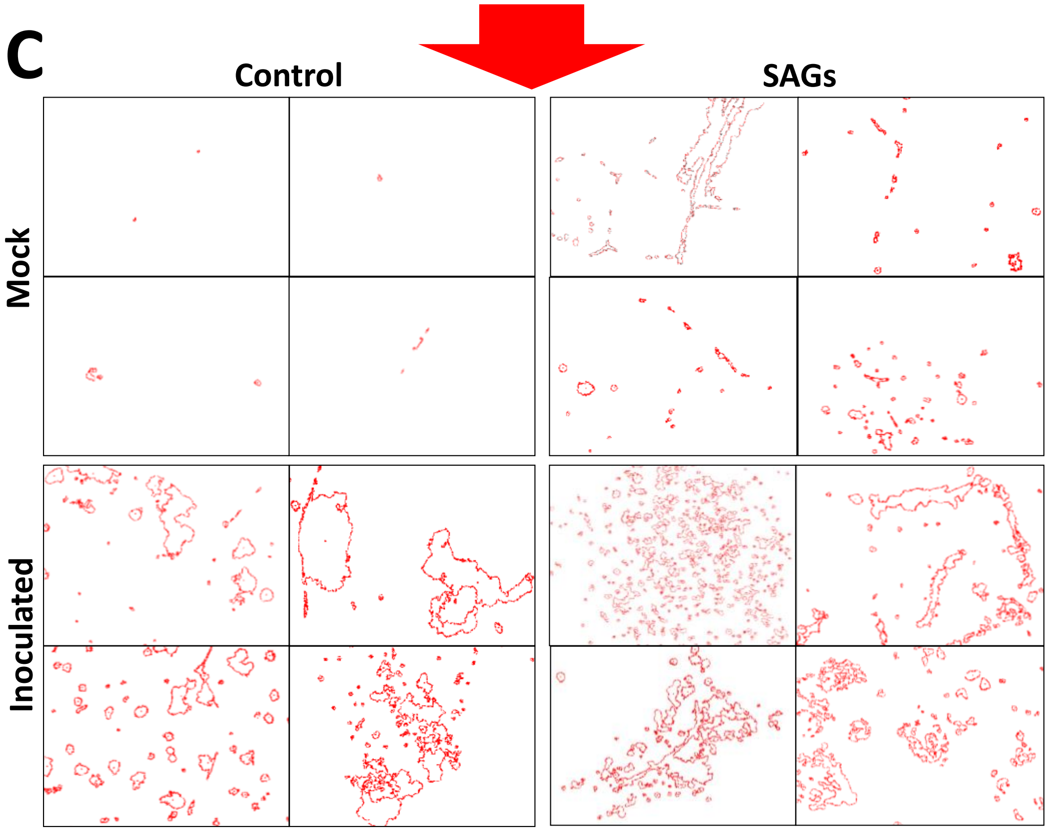
C
Control
SAGs
Mock
Inoculated

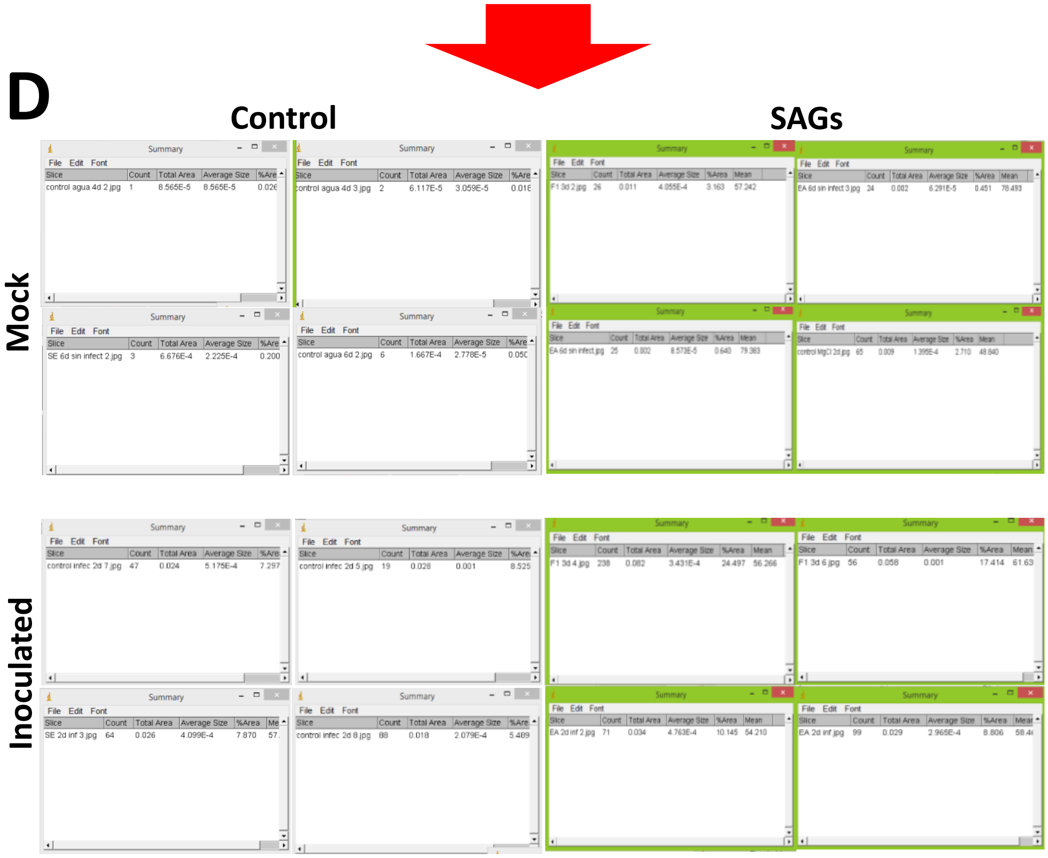
D
Control
SAGs
Mock
Inoculated

Supplementary Figure S4. Quantification of callose accumulation in leaves of SAGs (10ug/ml)- or water-treated plants (control) later inoculated or not inoculated (Mock) with the bacterial phytopathogen Pseudomonas viridiflava. Four representative pictures per treatment are shown. A) Callose deposition was visualized, under fluorescence microscope using UV light, as brilliant areas after aniline blue staining. Each image corresponds to a 25 mm2 leaf area. B) Fluorescence areas were detected using the image processing and analysis software Image J (red areas) to each microphotography previously converted to grayscale image. C) Detected brilliant areas in each image were extracted in a new image, and (D) calculated as Total Area (cm2) which results are shown in a new window denoted as Summary generated by the Image J software. These values were graphically visualized and statistically evaluated in Figure 2B.

Supplementary Figure S5

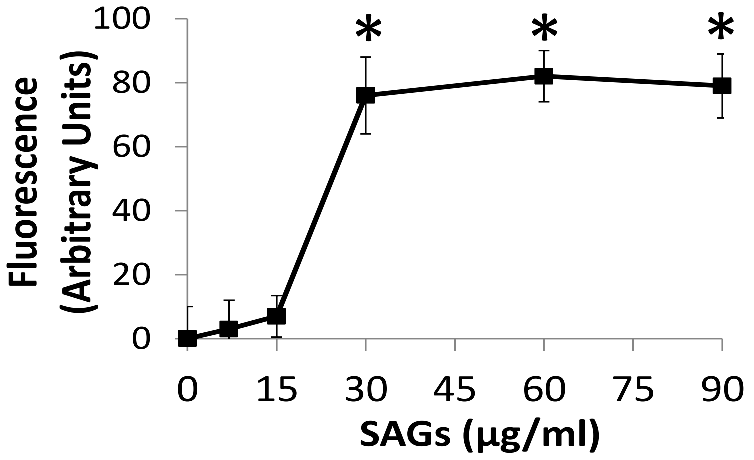

Supplementary Figure S5. Dose-dependent antimicrobial activity of SAGs measured as membrane permeability of C. michiganensis using the fluorescent dye diSC3(5). (*) Denote significantly difference respect to treatment without SAGs.

Supplementary Figure S6

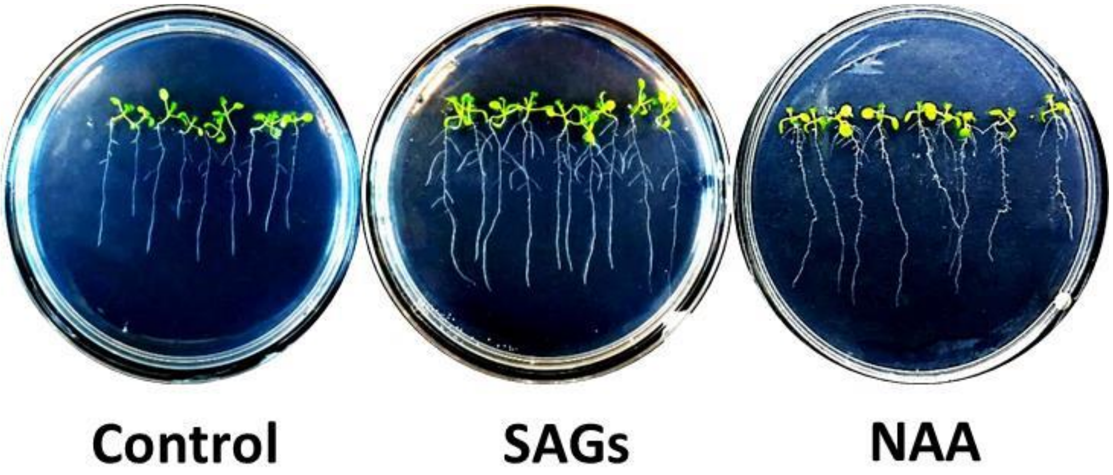

Supplementary Figure S6. Root growth stimulation of SAGs. (A) Phenotype of root development of Arabidopsis seedlings grown seven days on medium supplemented with 0.16ug/ml SAGs or 5ng/ml naphthalene acetic acid (NAA), and control plants grown on non-supplemented medium. Experiments were repeated three times using 10 plants for each experiment.
